# Metastatic renal cell carcinoma in the thyroid gland

**DOI:** 10.1002/ccr3.3088

**Published:** 2020-07-05

**Authors:** Mark M. Cruz, Gregory S. Schmidt, Jeptha T. Johnson, Thanh D. Hoang, Mohamed K. M. Shakir

**Affiliations:** ^1^ Division of Endocrinology Department of Medicine Walter Reed National Military Medical Center Bethesda Maryland USA; ^2^ Department of Pathology Walter Reed National Military Medical Center Bethesda Maryland USA

**Keywords:** fine‐needle biopsy, malignancy, PET‐CT, RCC, thyroid

## Abstract

Thyroid incidentalomas on FDG PET/CT are common with one‐third of focal uptakes caused by malignancies. Toxic nodules should be excluded. Ultrasound risk‐adapted systems can classify thyroid nodules and identify those who need biopsy.

## Graphical abstract

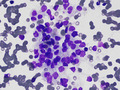

## 1 CLINICAL VIGNETTE

**FIGURE 1:**
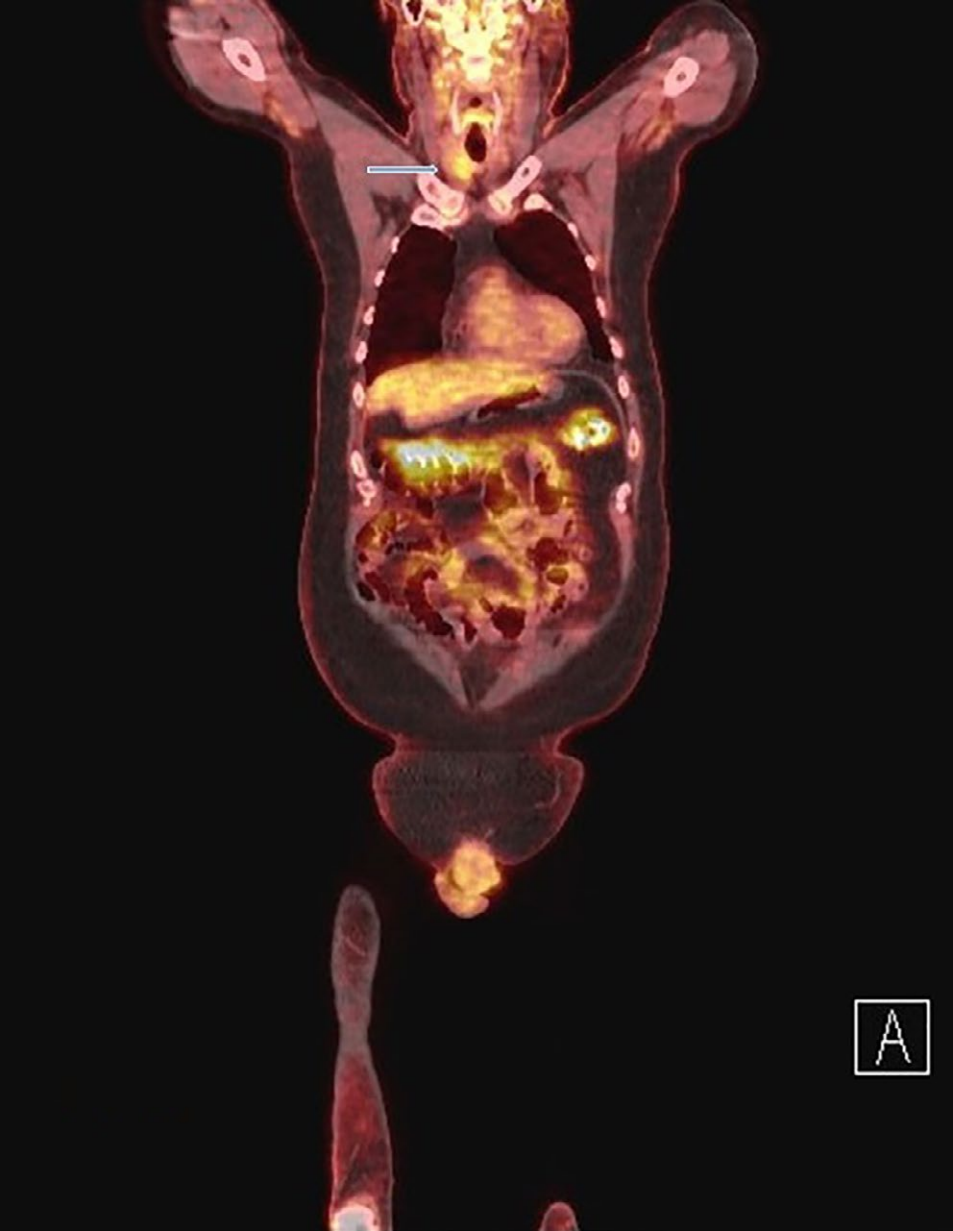
PET‐CT demonstrating an FDG‐avid right‐sided thyroid nodule with a standard uptake value of 4.58

**FIGURE 2:**
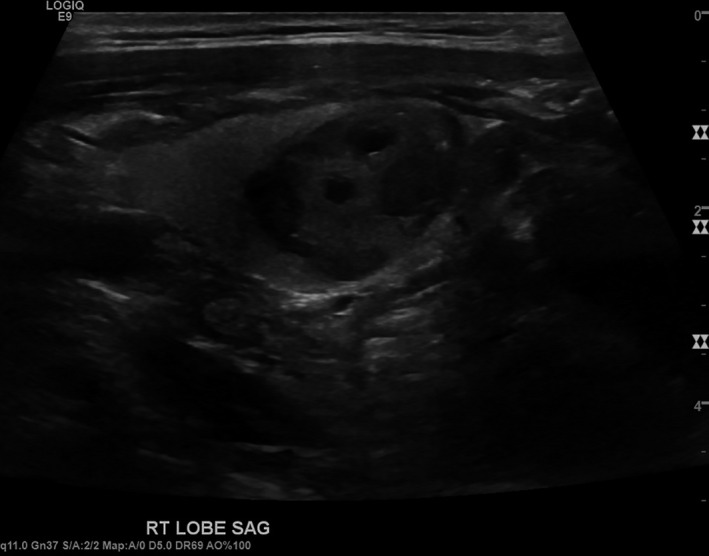
Thyroid ultrasound with a 2.2 cm hypoechoic nodule in the right middle lobe with faint microcalcifications

**FIGURE 3:**
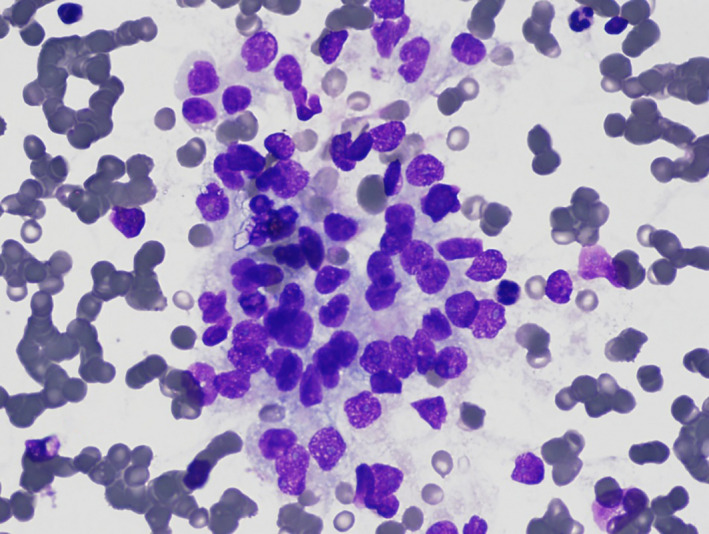
A cytologic smear of the FNA demonstrating cohesive groups of cells with large nuclei, irregular nuclear contours, granular chromatin with finely vacuolated cytoplasm “champagne bubble cytoplasm”

**FIGURE 4:**
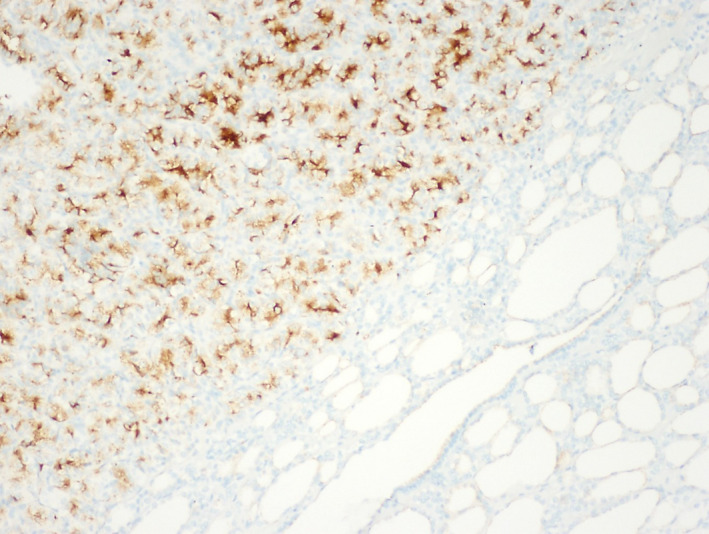
An immunohistochemistry with RCC and PAX8

An 83‐year‐old man presented for evaluation of non‐toxic multinodular goiter. Medical history was significant for metastatic renal cell carcinoma (RCC) to the hip diagnosed in 2013 with on‐going treatment with ipilimumab/nivolumab. A surveillance positron emission tomography‐computed tomography (PET/CT) demonstrated an fluorodeoxyglucose‐avid right‐sided thyroid nodule with a standard uptake value (SUV) of 4.58 (Figure 1). Thyroid ultrasound revealed a 2.2 cm hypoechoic solid nodule in the right mid‐lobe with faint microcalcifications, TI‐RADS 4 (Figure 2). The patient denied dysphagia, neck radiation, or family history of thyroid cancer. Thyroid function tests were normal. Fine‐needle aspiration (FNA) showed cohesive groups of cells with large nuclei, irregular nuclear contours, granular chromatin with “champagne bubble cytoplasm,” Bethesda category VI (Figure 3). Subsequent thyroidectomy specimen was positive staining for RCC marker and PAX8 by immunohistochemistry, confirming metastatic RCC (Figure 4).

Thyroid nodules are often incidentally found on imaging studies with an estimated 25% incidence on neck CT scan.^1^ The risk of malignancy in thyroid nodules identified on PET‐CT is reported 27.8%‐74.0%, and nodules with higher SUVs have a higher likelihood of malignancy.^1^ The overall incidence of metastatic disease to the thyroid gland is approximately 2% in autopsy series.^2^ The most common primary malignancy in cases of thyroid metastases is RCC followed in descending order by lung, gastrointestinal, and breast malignancies. In our patient, both PET‐avidity and presence of microcalcifications in the thyroid nodule suggested a high risk of malignancy.

This case highlights the importance of high clinical suspicion in patients with PET‐avid thyroid nodules, especially those with a history of malignancy. Such patients should undergo FNA to evaluate for malignancy given the high pretest probability.

## CONFLICT OF INTEREST

None to declare.

## AUTHOR CONTRIBUTIONS

MMC served as author. GSS: served as reviewer. JTJ: served as pathology reviewer. TDH: served as reviewer, editor and submitter. MKMS: served as reviewer. All authors were directly or indirectly involved in the care of the patient.

## ETHICAL APPROVAL

This manuscript has been cleared by the institutional review board. The patients have given written informed consent to publish the case (including publication images).

## DISCLAIMER

The views expressed in this article are those of the authors and do not reflect the official policy of the Department of Army/Navy/Air Force, Department of Defense, or the US Government.
